# Effects of Rice Straw Powder (RSP) Size and Pretreatment on Properties of FDM 3D-Printed RSP/Poly(lactic acid) Biocomposites

**DOI:** 10.3390/molecules26113234

**Published:** 2021-05-27

**Authors:** Wangwang Yu, Lili Dong, Wen Lei, Yuhan Zhou, Yongzhe Pu, Xi Zhang

**Affiliations:** 1 College of Science, Nanjing Forestry University, Nanjing 210037, China; yuww@niit.edu.cn (W.Y.); njfu001@21cn.com (L.D.); zyh990312@163.com (Y.Z.); puyongzhell@163.com (Y.P.); tougao0142@163.com (X.Z.); 2 School of Mechanical Engineering, Nanjing Vocational University of Industry Technology, Nanjing 210023, China

**Keywords:** poly(lactic acid) (PLA), rice straw powder (RSP), fused deposition modeling (FDM), biocomposite, pretreatment

## Abstract

To develop a new kind of environment-friendly composite filament for fused deposition modeling (FDM) 3D printing, rice straw powder (RSP)/poly(lactic acid) (PLA) biocomposites were FDM-3D-printed, and the effects of the particle size and pretreatment of RSP on the properties of RSP/PLA biocomposites were investigated. The results indicated that the 120-mesh RSP/PLA biocomposites (named 120#RSP/PLA) showed better performance than RSP/PLA biocomposites prepared with other RSP sizes. Infrared results showed that pretreatment of RSP by different methods was successful, and scanning electron microscopy indicated that composites prepared after pretreatment exhibited good interfacial compatibility due to a preferable binding force between fiber and matrix. When RSP was synergistically pretreated by alkaline and ultrasound, the composite exhibited a high tensile strength, tensile modulus, flexural strength, and flexural modulus of 58.59, 568.68, 90.32, and 3218.12 MPa, respectively, reflecting an increase of 31.19%, 16.48%, 18.75%, and 25.27%, respectively, compared with unmodified 120#RSP/PLA. Pretreatment of RSP also improved the thermal stability and hydrophobic properties, while reducing the water absorption of 120#RSP/PLA. This work is believed to provide highlights of the development of cost-effective biocomposite filaments and improvement of the properties of FDM parts.

## 1. Introduction

Three-dimensional printing, also known as additive manufacturing, is a new technology that offers more design freedom and it enables designers to create real objects based on a virtual computer model [1]. Three-dimensional printing creates numerous new possibilities for constructing geometries that cannot be realized by other methods, and it creates functional parts without requiring specialized tools and molds and also without involving any traditional cutting techniques [2,3]. When some parts with complex structures need to be manufactured in small batches or when unique pieces/prototypes are produced, 3D printing has great advantages over any conventional process [2].

There are different 3D printing techniques for fabricating polymers and composites, such as stereolithography, direct ink writing, inkjet printing, and fused deposition modeling (FDM). Of these, FDM is the most widely adopted technique for the fabrication of polymer composites and thermoplastics with low melting points [4,5], and a wide range of materials is available, from neat polymers to more recent composites, for FDM.

PLA, one kind of biodegradable polymer derived naturally from agricultural products, such as corn, rice, and sugar beets, has been explored in FDM. This is because PLA is an environment-friendly polymer with good stiffness, high strength, a low thermal expansion coefficient, and low elongation at break [1,6]. PLA reduces impact effects, does not adhere to the printed surface, and does not crack in large pieces. It shows good biocompatibility under physiological conditions and is biodegradable through hydrolytic degradation, as well as bioresorbable, with its end degradation product being metabolized through lactic acid cycles in vivo [7]. PLA is harder but melts at a lower temperature than ABS and has no health risk for humans when used in nonventilated areas. However, its high production cost limits the wider application of PLA’s FDM technology. 

Natural fibers are emerging as suitable reinforcing agents in polymeric matrices because of their availability at an affordable cost, renewable nature, and unique properties, including low specific gravity and biodegradability. Therefore, the feasible evaluation of natural fiber/PLA composites is of great importance for exploring feedstock for 3D printing applications. For this reason, some biocomposites have been FDM-3D-printed with PLA and natural fibers. Liu et al. [8] investigated the effect of printing orientation on the morphologies, mechanical properties, crystallization properties, and thermal stability of FDM-3D-printed sugarcane bagasse (SCB) fiber/PLA biocomposites. Dong et al. [9] printed mechanical testing specimens of PLA and PLA/wood fiber composites and investigated 3D printing parameters, including infill density, layer height, and the number of shells via design of experiments (DoE), and evaluated the effect of material type and the number of shells on the strength of specimens. Kariz et al. [10] investigated the effect of wood content in FDM filaments on the properties of 3D-printed parts. In addition to the aforementioned natural fibers, fibers such as cork [11], flax [12,13], thermomechanical pulp fibers [14], husk [15], energy grass (silvergrass, pennisetum, reed) [16], bamboo [17], and lignin [18] have also been used to complex with PLA to produce filaments for FDM 3D printing.

Rice straw, one of the plentiful and renewable natural fibers, is often disposed of by burning. On the one hand, burning rice straw results in serious air pollution and carbon emissions. On the other hand, a great deal of a natural resource is wasted. Actually, the lignocellulosic structure of rice straw containing natural composites is a potential alternative natural fiber in wood plastic composite processing [19,20]. However, it is seldom reported to be used to produce filaments for FDM 3D printing.

This paper focuses on the development of FDM-3D-printed PLA biocomposites that incorporate rice straw powder (RSP) to further advance the use of rice straw toward packaging and many other applications, enhance their biodegradability, and reduce costs of the products. The effects of RSP size on the morphologies and properties of printed specimens were first investigated, and then three kinds of pretreatment methods were evaluated to determine the improvements that these modifications provide to the printed RSP/PLA biocomposites. The mechanical properties, morphological behavior, as well as thermal properties of RSP/PLA biocomposites were investigated. Additional emphasis was placed on wettability studies and on the water absorption of RSP/PLA biocomposites.

## 2. Materials and Methods

### 2.1. Materials and Reagents

For this study, 3052D virgin poly(lactic acid) with a density of 1.24 g/cm^3^, a glassy transition temperature (Tg) between 55 and 60 °C, and a melting temperature (Tm) between 145 and 160 °C was obtained from American Nature Works Co. RSP (60, 120, 160, and 200 meshes) was prepared by grinding rice straw locally obtained in Nanjing City, China. Sodium hydroxide (NaOH), CP, was obtained from the Linfeng Chemical Reagent Co. Ltd., Shanghai, China.

### 2.2. Pretreatment of RSP 

#### 2.2.1. Alkali Pretreatment

RSP was soaked in 5 wt % NaOH solution (the mass ratio of RSP and NaOH solution was 1:20) for 8 h at room temperature. Then, the RSP solution was filtered, neutralized with 1 wt % acetic acid solution, and washed with distilled water. It was finally dried in an oven for 10 h at 105 °C. The dried fibers were then disintegrated into powder in a high-speed mill, sieved, and named A-RSP.

#### 2.2.2. Pretreatment with Ultrasound

RSP was dissolved in distilled water (the mass ratio of RSP and distilled water was 1:20), then treated at 20~30 °C for 30 min with ultrasonic waves whose output power was 600 W, and dried in an oven for 10 h at 105 °C. The dried fibers were then disintegrated into powder in a high-speed mill, sieved, and named U-RSP.

#### 2.2.3. Combination Pretreatment with Alkaline and Ultrasound

RSP was first modified by an alkali pretreatment process and dried, followed by modification with ultrasonic pretreatment, and named AU-RSP.

### 2.3. Preparation of FDM Filaments

RSP and PLA were weighed in a mass ratio of 1:99 and then dried for 12 h at 60 °C. FDM filaments were manufactured using a twin-screw extruder (KS-HXY; Kunshan Huanxinyang Electrical Equipment Co., Ltd., Suzhou, China), the RSP–PLA mixture was fed through a hopper to the extruder, and the heater was set at a temperature of 170 to 190 °C from hopper to die. The filament diameter was controlled within 1.75 ± 0.05 mm.

### 2.4. Specimen Manufacturing

First, the sample model file (STL file) for the tensile or flexural test was designed by computer-aided design according to the standard ISO 527-1 “Plastics—Determination of Tensile Properties—Part I: General Principle” (International Organization for Standardization, Geneva, Switzerland, 2012) or ISO 178 “Plastics—Determination of Flexural Properties” (International Organization for Standardization, Geneva, Switzerland, 2010), respectively. The model file was sliced and transformed into G-code using an open source software (Cura 15.04), and a series of corresponding printing parameters were set. All specimens were then manufactured using an FDM 3D printer (MOSHU S108; Hangzhou SHINING 3D Technology Co., Ltd., Hangzhou, China) fitted with a 0.4 mm nozzle in accordance with G-code; the printing parameters were 100% infill density, 0.2 mm layer thickness, 55 mm s^−1^ printing speed, 205 °C nozzle temperature, and 45 °C bed temperature. 

Composites were labeled 60#RSP/PLA, 120#RSP/PLA, 160#RSP/PLA, and 200#RSP/PLA when the sizes of RSP were 60, 120, 160, and 200 meshes, respectively, and composites were labeled A-RSP/PLA, U-RSP/PLA, and AU-RSP/PLA with the pretreatment methods of RSP corresponding to alkali, ultrasonic, and combination of alkali and ultrasonic, respectively.

### 2.5. Measurement and Characterization

#### 2.5.1. Determination of Mechanical Properties 

Tensile or flexural experiments were performed in accordance with ISO 527-1 or ISO 178 using a universal machine (E44.304; MTS Industrial Systems (China) Co., Ltd., Shenzhen, China) at a crosshead speed of 10 or 5 mm/min. The strength and modulus were evaluated as the average of at least 20 specimens.

#### 2.5.2. Morphology Analysis

The surface morphology of some tensile fractures of the printed specimens was observed and photographed by SEM (SU8010; Hitachi, Ltd., Hitachi, Japan) under an accelerating voltage of 3 kV in high-vacuum mode. The surfaces of the samples were sputter-coated with gold for 20 s to avoid sample charging during imaging by vacuum deposition using a sputter coater before SEM observation. 

#### 2.5.3. Thermogravimetric Analysis (TGA)

The thermal stability of the specimens was measured using a TG 209F1 analyzer (NETZSCH-Gerätebau GmbH, Selb, Germany). Approximately 5~12 mg of the samples was weighed and placed in an alumina ceramic crucible. Then, the samples were heated at a heating rate of 20 K/min from 25 to 550 °C under a N_2_ atmosphere. 

#### 2.5.4. Fourier Transform Infrared Spectroscopy (FTIR) 

RSP was mixed and milled with potassium bromide in a mass ratio of 1:100, and then tablet samples were prepared. Infrared spectra of the different samples were recorded using Frontier spectroscopy (VERTEX 70; Bruker Optics, Karlsruhe, Germany). The background was collected before any measurement. All measurements were performed with a spectrum range from 400 to 4000 cm^−1^, a scan speed of 1 cm/s, and a resolution of 4 cm^−1^. A total of 32 scans were collected, and the baseline was subtracted for correction. Bruker Spectrum software was used for data analysis.

#### 2.5.5. Wettability 

The contact angles of the 3D-printed specimens were measured with a contact angle instrument (DSA100; KRÜSS GmbH, Borsteler Chaussee, Germany) using a distilled water drop at room temperature. A 5 μL droplet of distilled water was dropped onto the specimen surface and kept for 15 s, and then the contact angles from the images were measured at different points; 10 specimens were used for each sample.

#### 2.5.6. Water Absorption 

Five 3D-printed specimens were immersed in distilled water at ambient temperature, taken out from the water after some time, and weighed in a balance with a precision of 0.1 mg after removing off the water on the surface with absorbent lint-free cloth. Next, the specimens were immersed in distilled water again. The percentage gain $x_{t}$ at time t, resulting from water uptake, was determined by Equation (1). This was repeated until the sample mass was almost constant.

(1) $$x_{t}=\frac{w_{t}-w_{0}}{w_{0}}\times 100\%,$$

where $w_{0}$ and $w_{t}$ denote, respectively, the sample weight before and after exposure to water. 

## 3. Results and Discussion

The properties of FDM-3D-printed specimens were affected by factors such as the printing parameters, the physical and chemical properties of raw materials, as well as the interfacial bonding between reinforcement and matrix. In this study, we focused on the effects of the particle size and pretreatment of RSP on the properties of FDM-3D-printed RSP/PLA biocompsosites.

### 3.1. Effect of RSP Size on Properties and Morphologies of 3D-Printed Specimens

#### 3.1.1. Mechanical Properties

**Figure 1:**
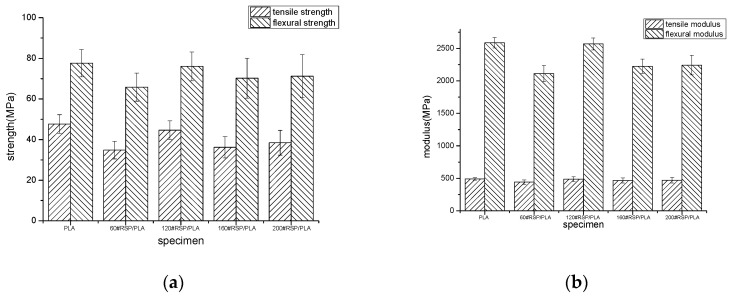
Mechanical properties of 3D-printed materials: (**a**) strength and (**b**) modulus.

Figure 1 shows the mechanical properties of 3D-printed PLA and the 60#RSP/PLA, 120#RSP/PLA, 160#RSP/PLA, and 200#RSP/PLA composites. It was found that both the flexural and the tensile properties of the 3D-printed specimens depend to a high degree on the particle size of RSP. Though adding RSP worsened the mechanical properties of PLA, the measured flexural strength, flexural modulus, tensile strength, and tensile modulus of 120#RSP/PLA were 76.06, 2568.96, 44.66, and 488.21 MPa, respectively, all being much higher than those of 60#RSP/PLA, 160#RSP/PLA, and 200#RSP/PLA. Compared with the 3D-printed PLA, the tensile strength, tensile modulus, flexural strength, and flexural modulus of 120#RSP/PLA were 97.99%, 99.31%, 93.67% and 99.10%, respectively, those of 3D-printed PLA.

Varying the particle size of RSP induces a dramatic change in the mechanical behavior of the 3D-printed samples (Figure 1a,b) compared to 3D-printed PLA. Such differences could be due to the geometry and thus the specific surface of the filler. RSP with a large particle size has a rougher surface, which is more difficult to uniformly disperse in the matrix, and there also exist more defects in RSP itself. As a result, the mechanical properties of the 3D-printed specimen become poorer because of the inherent uneven structure. On the contrary, RSP with smaller-size particles has a greater specific surface and less internal defects and can be dispersed in PLA more uniformly. In addition, interfacial bonding becomes stronger, and better mechanical properties can thus be obtained. However, when the size of RSP particles is too small, particle aggregation occurs easily and built-in stress may thus be produced in the 3D-printed specimen. In addition, the specific surface area is greater, and more cavities are created and act as internal defects, reducing the efficiency of the energy transferred within the composite; consequently, the mechanical properties of the specimen worsen. Here, 120 meshes may be a suitable size, and the mechanical properties of the 3D-printed 120#RSP/PLA are the best among all the printed RSP/PLA specimens, though a little poorer than those of 3D-printed pure PLA.

#### 3.1.2. Morphology

**Figure 2:**
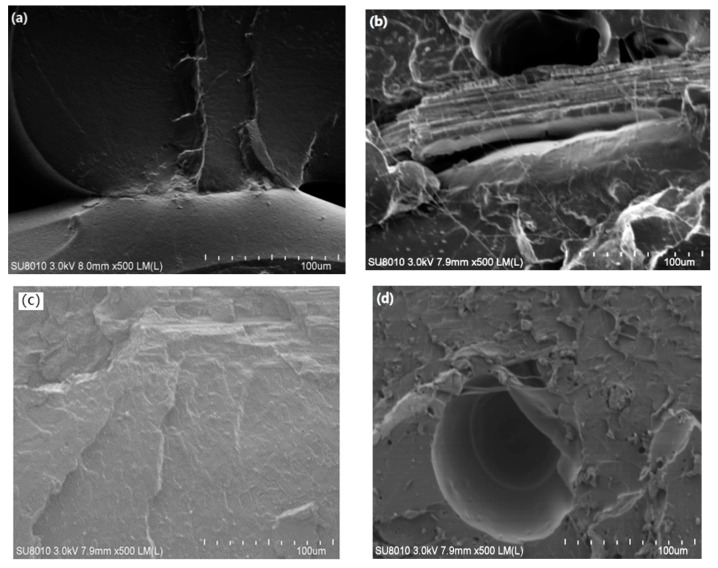
SEM micrograph of cross section of 3D-printed specimen of (**a**) raw PLA, (**b**) 60#RSP/PLA, (**c**) 120#RSP/PLA, and (**d**) 200#RSP/PLA. Scale bars in (**a**–**d**) indicate 100 μm.

Figure 2a–d shows SEM images of the fracture surfaces of PLA, 60#RSP/PLA, 120#RSP/PLA, and 200#RSP/PLA, respectively. It can be noted in Figure 2a that the fracture surface was smooth and no porosity could be observed for pure PLA. For the specimen with 120#RSP, there was good fiber/matrix adhesion as no fiber pullouts or gaps appeared between RSP and PLA. This indicated that good fiber/matrix adhesion was obtained. By comparing the three images in Figure 2b–d, it can be observed that there existed fiber pullouts and/or gaps between the fiber and the matrix when 60#RSP or 200#RSP was used, and there also was higher porosity, as shown by the SEM image of either 60#RSP/PLA or 200#RSP/PLA. In these situations, the bonding strength at the interface between RSP and PLA worsens. This phenomenon was consistent with the mechanical properties.

#### 3.1.3. Thermal Stability

**Figure 3:**
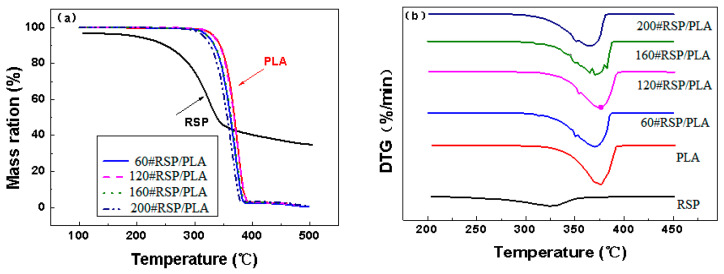
(**a**) TGA curves and (**b**) DTG curves for samples at 20 K/min in a nitrogen atmosphere.

Figure 3a,b illustrate the thermogravimetric (TGA) and first-derivative thermogravimetric (DTG) curves of the 3D-printed parts. The respective thermal properties for the main peaks are given in Table 1. It can be seen that pure RSP began to decompose at a much lower temperature than PLA, and the introduction of RSP into the PLA matrix accelerated the decomposition of PLA and thus caused weaker PLA thermal properties. This effect has been proved in previous studies [8,21]. In general, however, the decomposition behavior of the 120# RSP/PLA composite was much similar to that of pure PLA than the other composites. This phenomenon was also consistent with the mechanical properties.

The mechanical, morphological, and thermal results presented above all show a clear advantage of 120#RSP/PLA compared to 60#RSP/PLA, 160#RSP/PLA, or 200#RSP/PLA. Therefore, 120#RSP/PLA was taken as the research object, and the effects of RSP pretreatments on the morphologies and properties of 3D-printed 120#RSP/PLA composites were investigated. 

### 3.2. Effect of RSP Pretreatment on Morphologies and Properties of 3D-Printed Specimens

As reported in the literature [17], both the molding pressure and the shear rate for the FDM process are much lower than those for the common thermoplastic processing route, such as extrusion, compression, and injection. The material fails to develop high cohesion with strong interlayer interactions during 3D printing, which makes the 3D-printed biocomposites have poorer mechanical properties. Therefore, it is necessary to perform some pretreatments on the fiber to improve its cohesion with resin. In the second part of this work, RSP was alkali-pretreated, ultrasonic-pretreated, and combined-pretreated by alkaline and ultrasound, and the effects of the pretreatments on the morphologies and properties of FDM-3D-printed 120#RSP/PLA biocomposites were comparatively investigated.

#### 3.2.1. FTIR

**Figure 4:**
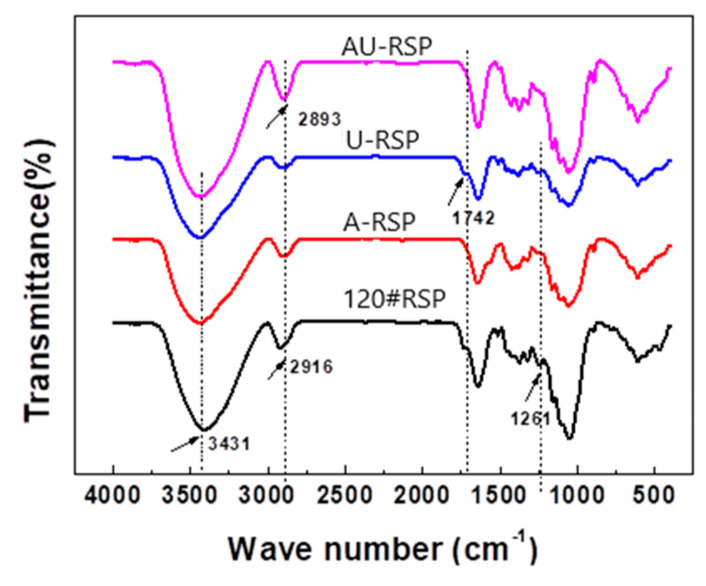
FTIR spectra of RSP before and after pretreatment.

FTIR spectra of RSP before and after pretreatment are shown in Figure 4. Obviously, the effect of pretreatment could be seen at the change in the peak position from 2916 cm^−1^ corresponding to the symmetric and asymmetric group CH stretching vibration (CH_2_) in lignin to 2893 cm^−1^, indicating that lignin was removed after RSP pretreatment. The peak at 1742 cm^−1^ corresponding to the C=O stretching vibration (nonconjugated) from hemicellulose and pectin could be observed in the FTIR spectra of the original RSP and U-RSP, but it disappeared in the FTIR spectra of A-RSP and AU-RSP, which indicated that alkali pretreatment can remove hemicellulose and pectin from RSP, while sole ultrasonic pretreatment cannot. Similarly, the peak at 1261 cm^−1^ corresponding to the C-O stretching vibration (nonconjugated) of the acetyl group in lignin [22] disappeared after alkali pretreatment. However, the peak around 1625 cm^−1^ assigned to the aromatic skeletal vibration from lignin and the peak at about 1320 cm^−1^ corresponding to characteristics of the S(syringyl) ring were observed in all samples. So, it can be concluded that some portions of amorphous components such as hemicellulose, pectin, and lignin can be removed from RSP by alkali pretreatment or combination pretreatment with alkali and ultrasonic, but not all, and that solely ultrasonic pretreatment cannot remove these components. Furthermore, the combined pretreatment with alkali and ultrasound could modify RSP much better than only alkali pretreatment.

#### 3.2.2. Mechanical Properties

**Figure 5:**
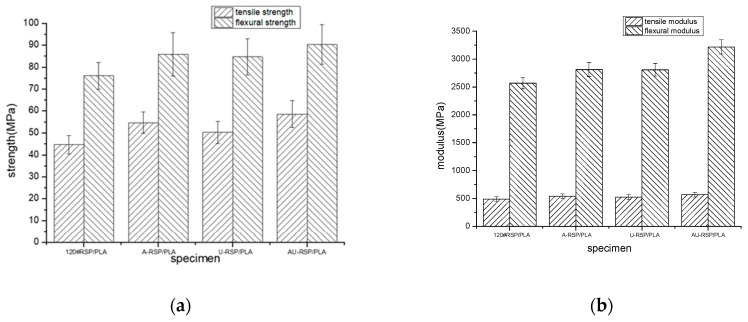
Mechanical properties of 3D-printed materials: (**a**) strength and (**b**) modulus.

**Figure 6:**
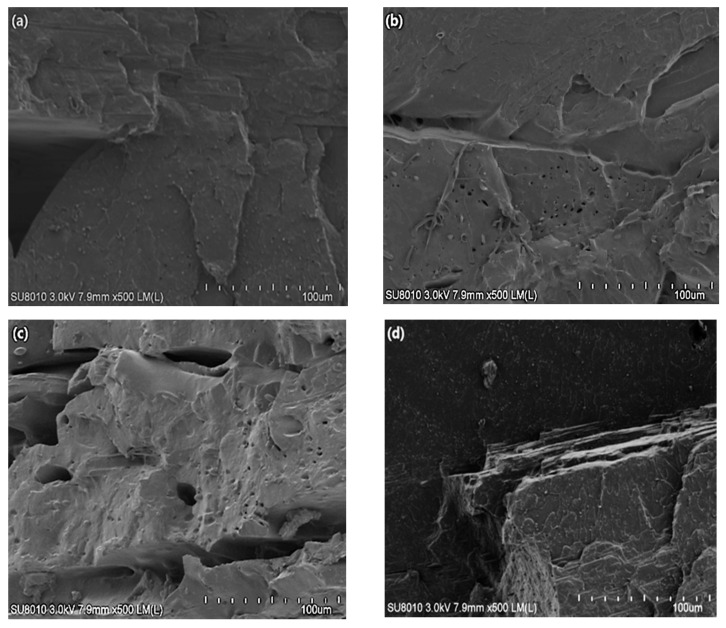
SEM micrograph of cross section of 3D-printed specimen of (**a**) RSP/PLA, (**b**) U-RSP/PLA/PLA, (**c**) A-RSP/PLA, and (**d**) AU-RSP/PLA. Scale bars in (**a**–**d**) indicate 100 μm.

The effects of RSP modification on the mechanical properties of the 3D-printed parts are demonstrated graphically in Figure 5. Overall, all the tensile and flexural strengths and moduli of the composites increased as RSP was pretreated, especially by combination pretreatment with alkali and ultrasonic; this was due to the improved interfacial adhesion between PLA and RSP, inducing the occurrence of fewer cavities at the interface between PLA and RSP, as evidenced in the following Figure 6. Especially for AU-RSP/PLA, its tensile strength and modulus were 58.59 and 568.68 MPa, respectively, and the flexural strength and modulus were 90.32 and 3218.12 MPa, respectively, reflecting a dramatic increase of around 31.19%, 16.48%, 18.75%, and 25.27%, respectively, from the unmodified 120#RSP/PLA.

#### 3.2.3. Morphology

The real cross sections of 3D-printed RSP/PLA specimens, depending on the pretreating of RSP, are presented in Figure 6a–d. Microscopic analysis showed that pretreating RSP with alkali or ultrasound caused smaller gaps than the untreated RSP/PLA biocomposite, though a few cavities or fiber pullout occurred, which enhanced the interfacial bonding between RSP and PLA. When RSP was pretreated with combined alkali and ultrasound, no gaps, cavities, or fiber pullout could be observed, which indicated that the bonding between RSP and PLA was strong and the modification was more effective than with alkali or ultrasonic pretreatment individually.

#### 3.2.4. Thermal Stability

**Figure 7:**
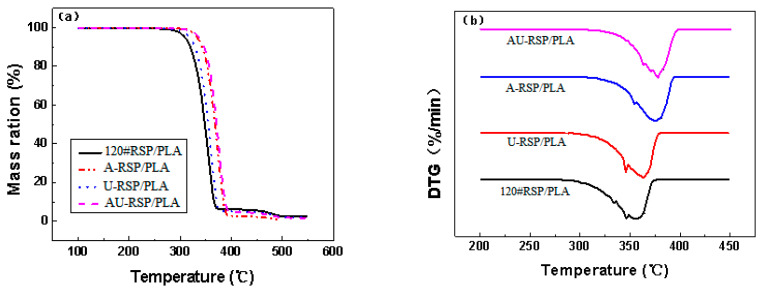
(**a**) TGA curves and (**b**) DTG curves for the samples at 20 K/min in a nitrogen atmosphere.

TGA and DTG curves for various RSP/PLA biocomposites are presented in Figure 7. The thermal stabilities of composites were characterized by a temperature at which the initial decomposition occurred (T_i_) and the temperature at which the maximum rate of weight loss occurred (T_p_). The results are summarized in Table 2. It was observed that the pretreatment of RSP increased the T_i_ and T_p_ of the composites, especially combined pretreatment of RSP with alkali and ultrasound increased T_i_ from 317.34 to 331.71 °C, a significant increase of 14.37 °C. This was attributed to the removal of low-molecular-weight substances in RSP (such as hemicellulose) in the composites that were found at a lower temperature. It was also observed that T_p_ increased slightly by 2.4 °C from 120#RSP/PLA to AU-RSP/PLA. All results mean that the thermal stability of the 3D-printed specimen was modified due to the better cohesion between RSP and PLA after pretreatment, which was also consistent with the results of FTIR analysis and mechanical property testing. 

#### 3.2.5. Wettability 

**Figure 8:**
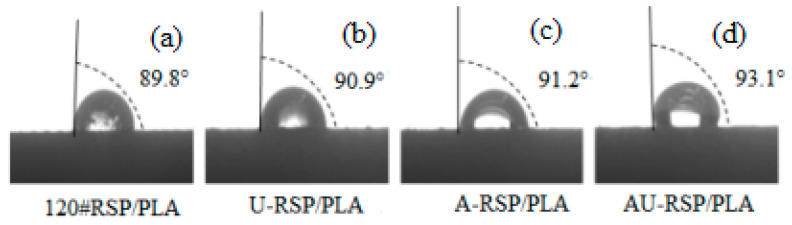
Contact angles of 3D-printed unmodified and modified RSP/PLA composites.

The shapes of water droplets for the tested surfaces of the specimens are shown in Figure 8a–d. The measured averaged contact angles are also given in the figure. The contact angle values of the specimens increased after RSP was pretreated. In other words, the wettability of the specimens decreased with the pretreatment of RSP. The contact angle value of 120#RSP/PLA was less than 90°. A contact angle less than 90° indicates that wetting of the surface is favorable, and the fluid will spread over a large area on the surface. However, the contact angle values of all the treated specimens, especially AU-RSP/PLA, were more than 90°, and the wettability of the 3D-printed specimen turned from the original hydrophilic to the hydrophobic state. However, there were no significant differences in the contact angle values between 120#RSP/PLA and U-RSP/PLA at all, and AU-RSP/PLA had the highest contact angle value. The test results of wettability further confirmed that the combined pretreatment with alkali and ultrasound has a synergistic effect on the fiber surface and the removal of amorphous materials such as lignin from RSP; consequently, the combination between matrix and fiber improves.

#### 3.2.6. Water Absorption

**Figure 9:**
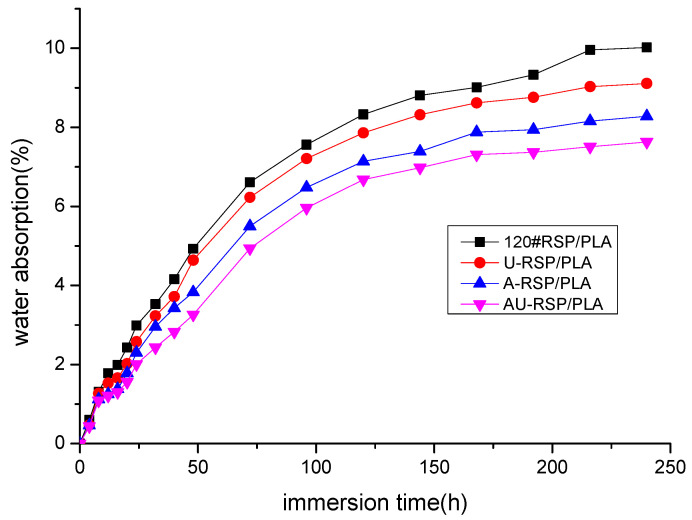
Water absorption curves for unmodified and modified RSP/PLA composites.

Water absorption curves based on experimental results were developed for each of the specimen types with varying modifications and immersion times (see Figure 9). The water absorption analysis results of the composite specimens showed that all the 3D-printed specimens with different pretreatments of RSP significantly differed from one another after immersion in water. This could be explained by the fact that the porosity of the specimens and the gaps among the filaments in the 3D-printed samples generally decreased when RSP was pretreated (Figure 6). These gaps could be filled with water during the long-term immersion in water, which increased the penetration of water into the specimens. Among all the specimens with the same immersion time, AU-RSP/PLA always absorbed the least water, which was due to no gaps, cavities, or fiber pullout observed for the AU-RSP/PLA specimen, as shown in Figure 6. On the contrary, 120#RSP/PLA always took the most water. 

## 4. Conclusions

In recent years, a renewed interest in FDM 3D printing has been seen, and more devices as well as materials have been developed. However, little systematic research has been undertaken on RSP-reinforced composite materials, especially regarding the effects of particle size and pretreatment of RSP on the properties of 3D-printed specimens. In the present study, RSP/PLA biocomposites were FDM-3D-printed, and the effects of particle size and pretreatment of RSP on the properties of the RSP/PLA biocomposites were investigated. The following conclusions are drawn:

1. The RSP/PLA biocomposite reinforced with RSP of 120 meshes showed a clear advantage compared to those prepared with other RSP sizes. Its flexural strength, flexural modulus, tensile strength, and tensile modulus were 76.06, 2568.96, 44.66, and 488.21 MPa, respectively, which were 97.99%, 99.31%, 93.67%, and 99.10% those of the 3D-printed pure PLA. The onset and peak temperatures of 120#RSP/PLA during thermal degradation were both greater than those of the other composites.
2. Alkali pretreatment, pretreatment with ultrasound, and combination pretreatment with alkali and ultrasonic were all shown to improve the overall performance of the composites, and combination pretreatment with alkali and ultrasonic exhibited the most apparent improvement in the properties, which improved interfacial bonding between RSP and PLA and simultaneously reduced the water absorption of the RSP/PLA biocomposites, enhancing the properties of the composites.

Overall, the RSP/PLA biocomposites developed in this study have adequate mechanical properties and thermal stability. Their hydrophobicity and water uptake properties can be improved by pretreatment of RSP; thus they show great promising potential for 3D printing applications. 

## Figures and Tables

**Table 1:** Thermal decomposition parameters for 3D-printed PLA and RSP/PLA composites in a nitrogen atmosphere.

Specimen	Ti/°C	TP/°C	Weight Residue at 550 °C/%
RSP	285.71	324.9	33.75
PLA	326.01	375.4	0.58
60#RSP/PLA	311.43	369.4	0.42
120#RSP/PLA	317.34	375.1	0.88
160#RSP/PLA	314.28	368.8	1.01
200#RSP/PLA	308.57	363.1	0.71

**Table 2:** Thermal decomposition parameters for 3D-printed unmodified and modified RSP/PLA composites in a nitrogen atmosphere.

Specimen	Ti/°C	TP/°C	Weight Residue at 550 °C/%
120#RSP/PLA	317.34	375.1	0.88
U-RSP/PLA	322.86	376.7	0.42
A-RSP/PLA	325.43	376.3	1.01
AU-RSP/PLA	331.71	377.5	0.71

## Sample Availability

Samples of RSP/PLA filament and printed specimens are available from the authors.
